# Effect of surgical experience on imageless computer-assisted femoral component positioning in hip resurfacing – a preclinical study

**DOI:** 10.1186/s40001-015-0086-8

**Published:** 2015-02-24

**Authors:** Maik Stiehler, Jens Goronzy, Stephan Kirschner, Albrecht Hartmann, Torsten Schäfer, Klaus-Peter Günther

**Affiliations:** University Centre for Orthopaedics & Trauma Surgery, University Hospital Carl Gustav Carus at Technische Universität Dresden, Fetscherstr. 74, Building 29, D-01307 Dresden, Germany; Department of Orthopaedics, St. Vincentius Clinic, Steinhäuserstrasse 18, 76135 Karlsruhe, Germany; Dermatological Practice, Kirchplatz 3, 87059 Immenstadt, Germany

**Keywords:** Computer-assisted surgery, Hip resurfacing, Navigation

## Abstract

**Background:**

The clinical outcome of hip resurfacing (HR) as a demanding surgical technique associated with a substantial learning curve depends on the position of the femoral component. The aim of the study was to investigate the effects of the level of surgical experience on computer-assisted imageless navigation concerning precision of femoral component positioning, notching, and oversizing rate, as well as operative time.

**Methods:**

Three surgeons with different levels of experience in both HR and computer-assisted surgery (CAS) prepared the femoral heads of 54 synthetic femurs using the Durom^TM^ Hip Resurfacing (Zimmer, Warsaw, IN, USA) system. Each surgeon prepared a total of 18 proximal femurs using the Navitrack® system (ORTHOsoft Inc., Montreal, Canada) or the conventional free-hand Durom^TM^ K-wire positioning jig. The differences between planned and postoperative stem shaft angle (SSA) and anteversion angle in standardized x-rays were measured and the operative time, not including the time for calibrating the CAS-system, was documented. Notching was evaluated by the three surgeons in a randomized manner. Oversizing was determined by the difference of the preoperative determined cap and the cap size advised by the CAS-system.

**Results:**

CAS significantly reduced the overall mean deviation between planned and postoperative SSA in comparison with the conventional procedure (mean ± SD, 1 ± 1.7° vs. 7.4 ± 4.4°, *P* <0.01) regardless of the surgeon’s level of experience. The incidence of either varus or valgus SSA deviations exceeding 5° were 1/27 for CAS and 15/27 for the conventional method, respectively (*P* <0.001), corresponding to a reduction by 97%. Using CAS, the rate of notching was reduced by 100%.

**Conclusions:**

The accuracy of femoral HR component orientation is significantly increased by use of CAS regardless of the surgeon’s level of experience in our preclinical study. Thus, imageless computer-assisted navigation can be a valuable tool to improve implant positioning in HR for surgeons at any stage of their learning curve.

## Background

Hip resurfacing (HR) can be regarded as a femoral bone stock-conserving joint replacement alternative for adequately selected patients with encouraging mid-term results [1,2]. Major short-term risks include periprosthetic femoral neck fracture and early aseptic loosening of the femoral component [3-5]. The clinical outcome of HR as a demanding surgical technique associated with a substantial learning curve is dependent on the position of the femoral component. Previous studies have shown that both unfavorable positioning of the prosthesis and femoral neck notching may facilitate femoral neck fractures and bone/implant impingement as well as complications related to increased metal wear [6]. In case of a malpositioned HR implant, wear and corrosion may lead to a release of metal products into surrounding tissue and body fluids as well as internal organs. Metal accumulation may result in local adverse reactions to metal debris [7] and potentially induce systemic adverse effects [8,9]. Some devices have already been officially withdrawn from the market and others are no longer available.

In light of the increasing body of evidence suggesting an association of unfavorable HR component orientation and metal debris-induced local tissue reactions and systemic side effects, safe and reliable tools which can help achieveing a correct femoral component position are key for a successful long-term outcome in HR.

The use of computer assisted surgery (CAS) has shown an improvement in the accuracy of implant positioning in total knee and conventional total hip replacement (THR) [10-12]. This study set out to investigate the effect of surgical experience on the precision of femoral component orientation by CAS.

## Methods

### Study setup

Three surgeons with different levels of experience in both HR and CAS prepared the femoral heads of 54 Sawbone® samples (Sawbones® Europe AB, Malmö, Sweden) using the DUROM® Hip Resurfacing system (Zimmer, Warsaw, IN, USA). Each surgeon operated on a total of 18 synthetic femurs, of which 9 were prepared conventionally and by CAS, respectively. For image-free CAS of the femoral component, the CAS Durom® Hip Resurfacing and Navitrack® Surgical Navigation System (Zimmer CTFree navigation system software; Orthosoft, Montreal, Canada) was used according to the manufacturers’ recommendations. At the time of the onset of the study, surgeon A had performed less than 20 stem-based and no HR procedures. He had neither experience in CAS for either total knee or hip arthroplasty. Prior to this study, he was trained in CAS and conventional HR technique by an experienced surgeon both assisting and performing each procedure on two Sawbone® models. Surgeon B was beyond the learning curve of conventional HR (43 procedures) and of CAS in total knee arthroplasty (120 procedures), but within the learning curve of CAS in HR prior to the onset of the study. Surgeon C is a high-volume surgeon in both conventional (183 procedures) and navigated (58 procedures) HR. Table 1 summarizes the level of surgical education of the three surgeons that participated in this study.

**Table 1:** **Level of experience of the three surgeons that participated in this study**

**Surgeon**	**Conventional HR**	**CAS, TKA**	**CAS, HR**
A	Within learning curve	Within learning curve	Within learning curve
B	Beyond learning curve	Beyond learning curve	Within learning curve
C	Beyond learning curve	Beyond learning curve	Beyond learning curve

HR, Hip resurfacing; CAS, Computer-assisted surgery; TKA, Total knee arthroplasty.

To show possible advantages for CAS in precision, deviations between post- and preoperative center collum diaphyseal (CCD) and anteversion angles were measured. Furthermore, to determine intraoperative complications inhibited by use of CAS, intraoperative warnings issued by the computer system and notching events at the head-neck junction were documented. Moreover, procedure time being a potential drawback of CAS was analyzed group-wise and the smallest safe component size recommended by the CAS system was recorded to detect errors in terms of femoral component oversizing induced by CAS. A possible learning curve, defined as improvement of cap placement, reduction of operative time, less oversizing, or femoral neck notching with increasing experience during the procedures, was documented.

### Navigation system

**Figure 1:**
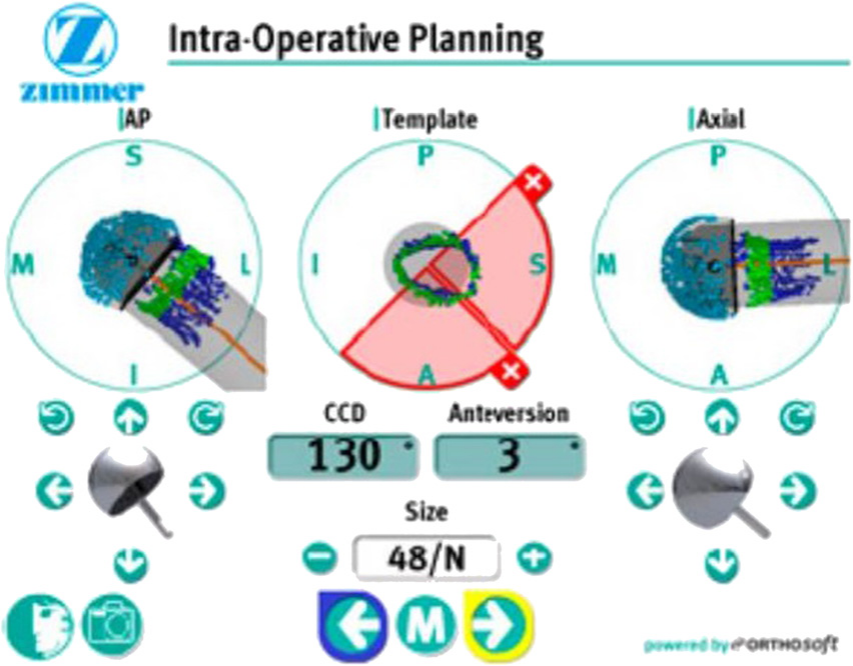
**Screenshot from the Navitrack® Software: The left (axial) and the right (ap) illustration show the digitalized femur.** The template in the middle demonstrates risks for possible notching in each quadrant of the neck (red quadrants).

The CAS Durom® Hip Resurfacing and Navitrack® Surgical Navigation system is a three-dimensional (3D), infrared-based, computer-assisted image-free navigation system providing data on the position of both the femoral and acetabular component intraoperatively during the HR procedure. The system calculates the motion axes of the hip joint from user-identified anatomical landmarks, i.e., piriformis fossa, femoral medial and lateral epicondyles, and lateral and medial malleolus. In addition, a 3D model of the femoral head and neck is digitized. The exact position of the implant in relation to the bone is then presented in real-time. Figure 1 shows the intra-operative screen shot of the Navitrack® planning software demonstrating digitized points on the femoral head and neck in anterior-posterior (AP), the template, the axial plane, the CCD and anteversion angles, as well as component size.

### Sawbone® samples

**Figure 2:**
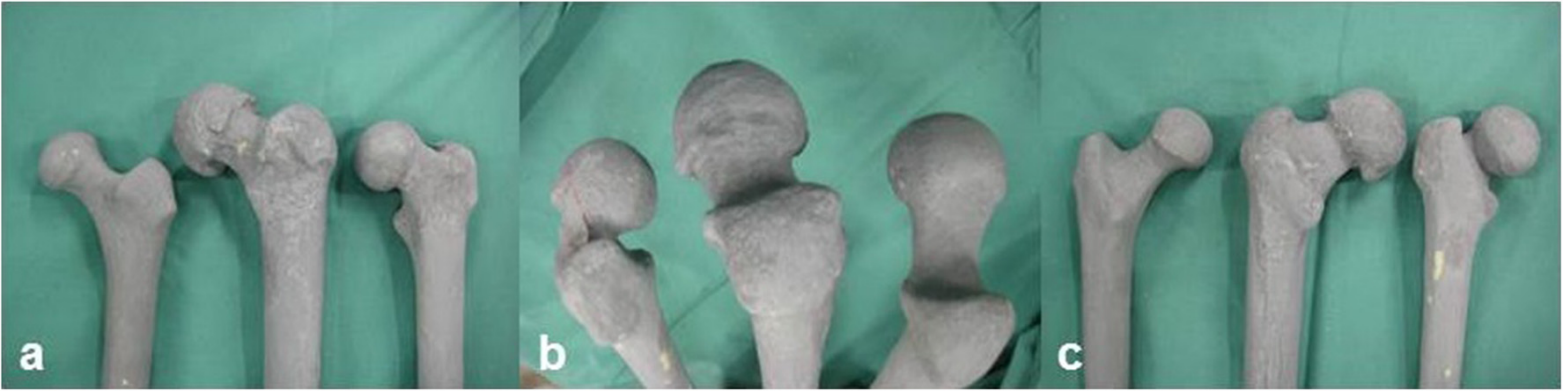
**Head/neck morphology of the three types of synthetic femurs used in this study.** Anterior **(a)**, top **(b)**, and posterior **(c)** view: normal configuration, osteophytes, slipped epiphysis (right to left).

**Figure 3:**
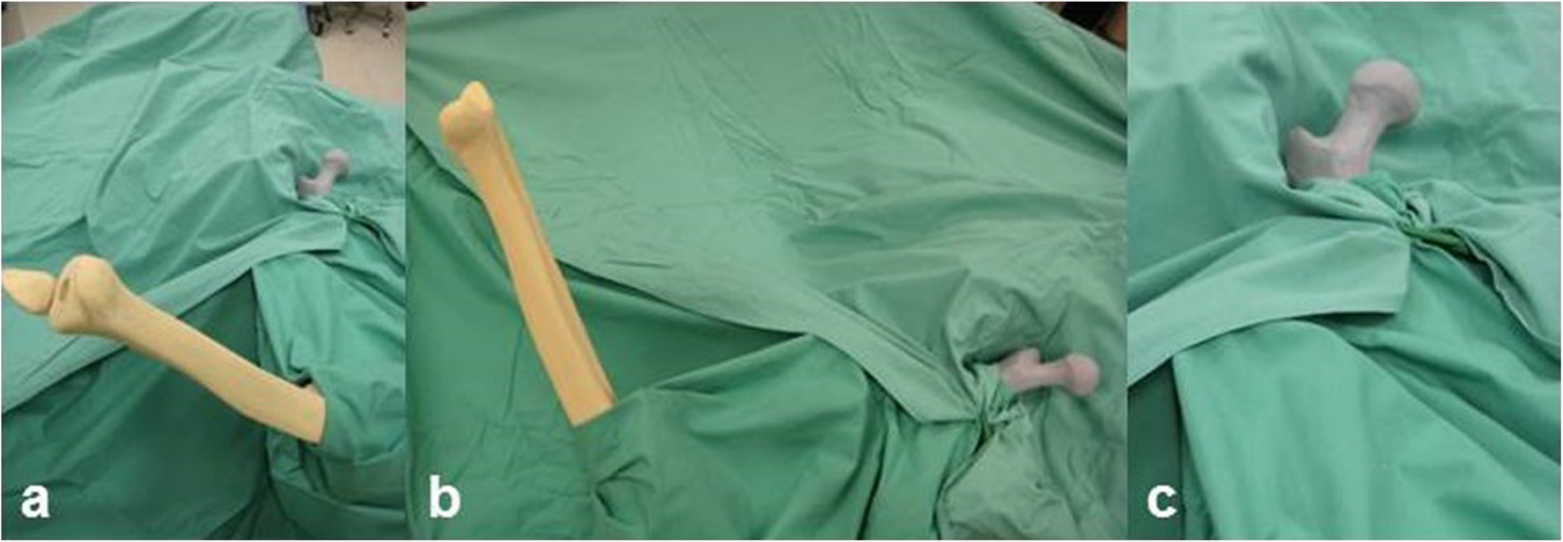
**Experimental setup mimicking the position of the proximal femur after exposure by dorsal approach during hip resurfacing procedure.** Overview **(a)**, side view **(b)**, and close up **(c)**.

Three morphologically different types of left Sawbone® femurs (“physiological” #1100, “osteoarthritis” #1197, “slipped capital epiphysis (SCFE)” #1161, Figure 2) were used for this study. The proximal halves of the specimens were painted with radiopaque colour (Genius Pro, Germany, Cosefeld) in order to allow an enhanced radiographic evaluation before and after intervention. During intervention the femurs were mounted onto a clamp (Sawbones®: universal bone clamp #1605) and positioned to mimic the intraoperative situation (Figure 3). Furthermore, the femurs were covered by drapes in a standardized manner only leaving the proximal medial part of the specimens (including the lesser trochanter, the piriformis fossa, the femoral neck, and the femoral head) visible to the surgeon, thereby avoiding the risk of orientation along the femoral diaphysis. The surgeons followed the manufacturers’ guidelines for HR and CAS.

A tibia/fibula Sawbone® combination (#1144 used with the osteoarthritic femur and #1144-1 used for both the physiological and the SCFE femur) was attached to each of the three different femoral Sawbones® resulting in 90° knee flexion. To reproduce the intraoperative position of the proximal femur, the Sawbone® models were mounted onto a clamp in internal rotation.The first step for both conventional and CAS technique was to measure the widest part of the femoral neck with a slide ruler to determine the adequate femoral component size.

For conventional HR, the surgeon removed approximately 6 mm of the apical femoral bone stock using the femoral head planner guided by a K-wire placed in line with the midpoint of the femoral neck in both planes. Then the centering jig was attached and adjusted with the help of a stylus simulating the chosen size of the cylindrical reamer to determine possible notching. After having achieved the optimal placement of the jig, the surgeons placed the definitive guide wire, which was then overdrilled. Subsequently, the guide wire/drill was replaced by the guide rode used for reaming with the determined size. The femoral head was cut to the correct depth using the femoral head planer inserted over the guide rod.

For CAS, the surgeons fixed the femoral reference near the intertrochanteric line of the femur. The femoral axis was calculated by digitizing the five following landmarks: piriformis fossa, medial and lateral epicondyle as well as medial and lateral malleolus with the knee flexed at 90°. Subsequently, the pointer was moved around the head and neck until sufficient digitized points were collected to reconstruct a 3D model. Implant size, CCD, and anteversion angles were chosen with the help of the calculated 3D model aiming to prevent notching. The most optimal entry point for the K-wire was calculated and the proposed position was maintained with the help of the software’s orientation feedback throughout the drilling procedure. As for the conventional procedure, the K-wire was overdrilled and replaced by the guide rod. The correct CCD angle was constantly controlled by the navigation software during reaming with the cylindrical reamer. Finally, the surgeons marked the head-neck junction at the level where the mouth of the femoral implant should be positioned using the navigation pointer and the femoral head was reamed guided by the CAS system to avoid excessive removal of femoral bone stock.

### Radiographic evaluation

**Figure 4:**
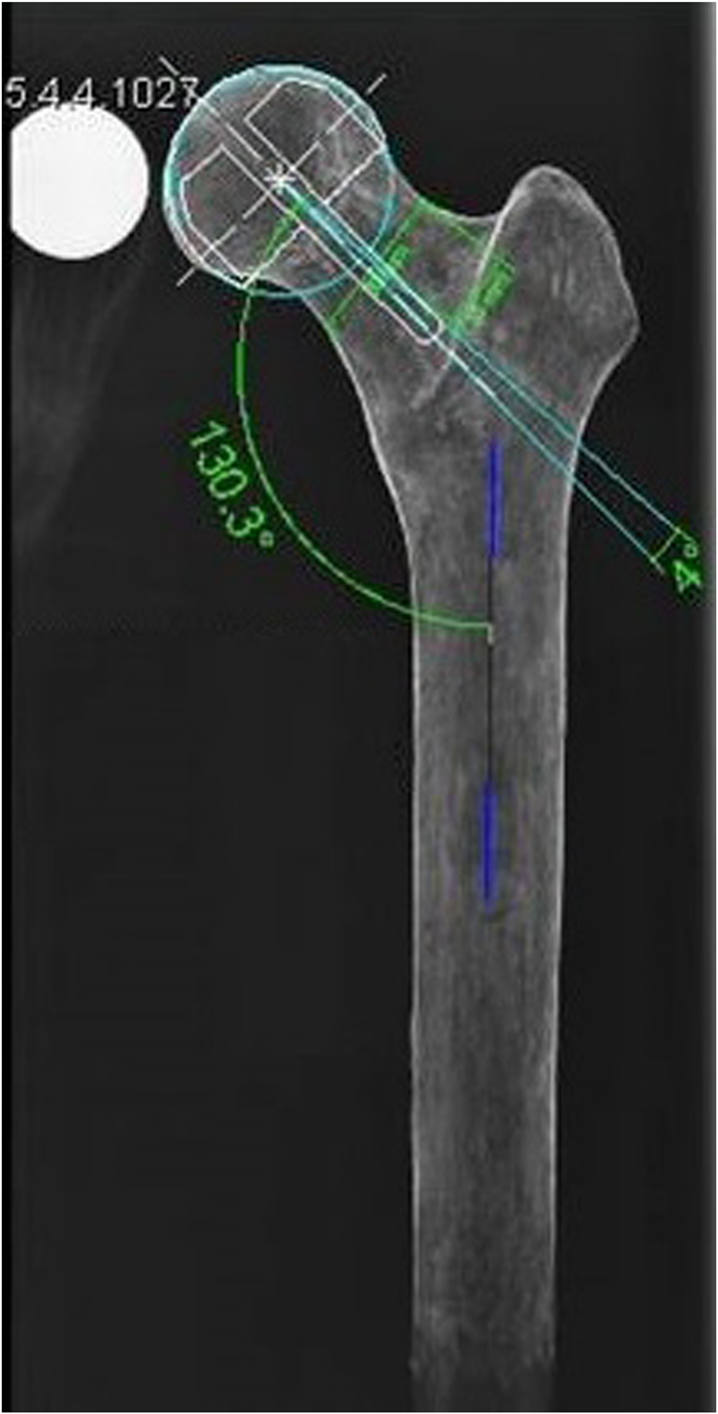
**Representative X-ray image including CCD angle (here 130.3°), planned position (here stem shaft angle 134°), and size (here “44”) of the femoral component.**

Before intervention, CCD and neck anteversion angles of each type of bone sample used were measured using mediCAD® imaging software Version 2.20 Hectec GmbH, Niederviehbach, Germany). Moreover, the planned CCD angles and implant sizes for each type of Sawbone® femur were determined (Table 2). For this, X-rays of the specimens included a 30 mm metallic scaling ball placed with its center at the level of the femoral head center. In all three bone types used, the planned stem axis was chosen to be the line running from the center of the femoral head to the intersection of the rectangular line to the diaphysis through the middle of the lesser trochanter and the lateral cortex. Figure 4 exemplifies an X-ray with physiological and planned CCD angles and the planned size of the femoral component plotted.

**Table 2:** **Center collum diaphysis (CCD) and neck antetorsion angle of the three types of synthetic femurs used in this study were measured from anteroposterior and axial Lauenstein X-ray scans, respectively**

	**Normal**	**Osteophytes**	**Slipped epiphysis**
**CCD angle**	130°	115°	116°
**Neck antetorsion angle**	6°	3°	21°
**Planned CCD angle**	134°	133°	130°
**Planned antetorsion angle**	6°	5°	-9°
**Planned implant size**	44	48	44

The planned stem shaft angle and implant size were determined using mediCAD® imaging software.

To obtain comparable pre- and postoperative X-ray images, each AP radiograph was taken on a planar X-ray table in a standardized manner. A reproducible axial radiograph was obtained using a custom-made Lauenstein rack, built for each of the three morphologically different synthetic bone types. Post-interventionally, a 75 mm long metal rod was inserted into the drilled canal for X-ray evaluation, and the achieved CCD and antetorsion angles were calculated. To measure the precision of cap placement, the preoperatively planed and the postoperatively achieved stem shaft angle (SSA) as well as the preoperatively planed and postoperatively achieved antetorsion angle were compared. The incidence of deviations exceeding 5° in the frontal and axial plane were defined as outliers.

### Intra- and postoperative evaluation

The procedure time was documented. The starting point for the conventional procedure was drilling the first K-wire and ended after the planar reaming. Measuring the time using CAS started with applying the femoral reference and ended after the planar reaming. The time needed for assembling and precalibrating the CAS system was not included in the recorded time as this step is usually accomplished by a qualified assistant rather than by the surgeon. To evaluate possible oversizing using CAS, notching warning events by the CAS software were documented. Furthermore, the smallest component size without any notching warnings was recorded. Notching was evaluated postoperatively by the three surgeons blinded to the synthetic femur samples. Superior, inferior, anterior, or posterior notching events were noted.

### Statistics

Differences between the planned and the achieved CCD and antetorsion angles were calculated. If not otherwise stated, data are presented as mean ± standard deviation of the mean. Intra-individual differences in deviation between planned and achieved angles were compared between the surgeons using the χ^2^ test or Fisher-Yates test, respectively. Furthermore, for selected variables a regression analysis was conducted to evaluate possible confounding parameters (i.e., surgeon, notching event, outlier, procedure time, bone morphology, CAS). Static measurements were performed with SPSS 17.

## Results

### Precision and deviation

**Figure 5:**
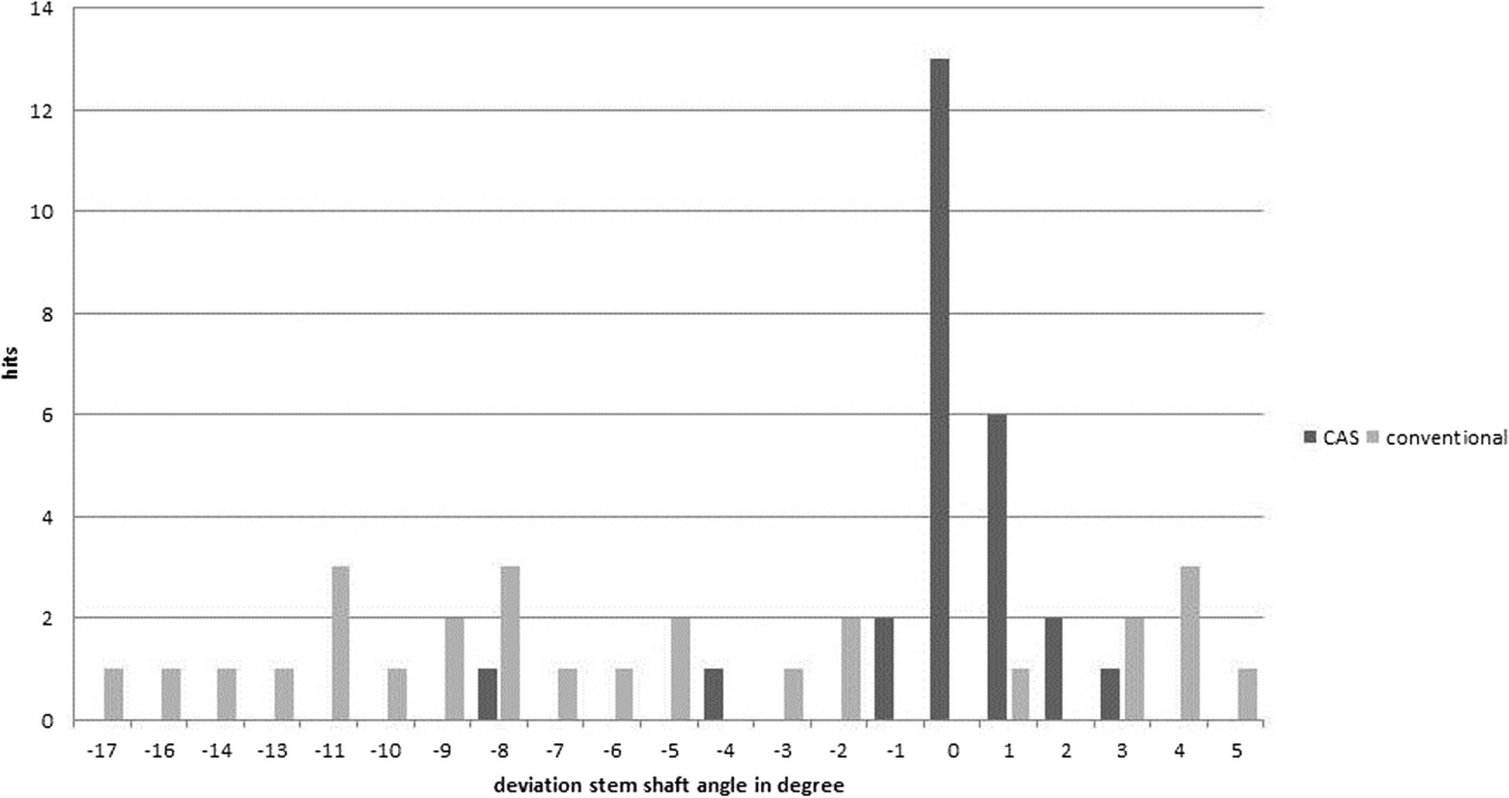
**Deviation between planned and achieved stem shaft angle in degree comparing conventional jig and computer-assisted surgery.**

Table 2 compares the results of using a conventional jig and CAS. In the CAS group, the overall deviation between planned and postoperative SSA angles was significantly reduced regardless of the surgeon’s level of experience as compared with the conventional group (1 ± 1.7° vs. 7.4 ± 4.4°, *P* <0.01; Figure 5). A regression analysis demonstrated that the only factor significantly improving precision was CAS, whereas neither level of experience and learning curve, nor the secondary outcome parameters procedure time and bone morphology showed any relevant influence on the precision in the frontal plane. Outliers in the AP plane were reduced by the use of CAS by 97%. While in the conventional group 15 (55.6%) outliers occurred, only 1 outlier (3.7%) was observed in the CAS group. A regression analysis evaluating the same parameters as mentioned for the comparison of the deviation between the planned and postoperative SSA angles showed that only CAS had a significant influence on reducing positional outliers.

In the conventional group, 20 (74.1%) guide rods were placed in varus and 7 (25.9%) in valgus position with mean deviations of 8.8 ± 4.3° and 3.4 ± 1.3°, respectively. Using CAS, 4 (14.8%) guide rods were placed in exactly (decimal step limit) the planned AP projection, whereas 13 (48.1%) with varus and and 10 (37.0%) with valgus alignment occurred with mean deviations of 3.5 ± 3.3° and 1.4 ± 0.7°, respectively. All outliers occurred in varus deviation. In the axial plane, the deviation between planned and postoperative angles was slightly reduced by CAS (3.9 ± 2.9° vs. 5.1 ± 3.1°, *P* = 0.13). As observed for the frontal projection, fewer outliers occurred in the CAS group as compared with the conventional group (n = 8, 29.6% vs. n = 12, 44.4%).

All three surgeons achieved a more favorable precision using CAS as shown in Table 3. In the frontal plane, the deviation was significantly reduced by the use of CAS regardless of the level of experience. The less experienced surgeon (A), in particular, improved in terms of reducing the deviation of 11.1 ± 4.4° to 1.0 ± 1.1° (*P* <0.01) and prevented any outliers (n = 8, 88.9% vs. n = 0, 0%; *P* <0.01). In addition, the more experienced surgeons B (3.6 ± 1.5° vs. 1.6 ± 2.7°; *P* <0.1) and C (7.4 ± 3.1° vs. 0.6 ± 0.5°; *P* <0.01) achieved a statistically significant superior precision using CAS. The most experienced surgeon (C) completely avoided outliers by use of CAS as compared to conventional implant positioning (n = 0, 0% vs. n = 6, 66.7%; *P* <0.01). Surgeon B generated one outlier (11.1%) in both CAS and conventional group.

**Table 3:** **Intraoperative and postoperative radiological data comparing the procedure using a conventional jig and computer-assisted surgery**

	**Conventional**	**Computer-assisted surgery**	***P*** **value**
Time (min)	14 ± 5 (7–29)	18 ± 6 (11–33)	0.028
**Frontal plane**			
Deviation (°)	7.4 ± 4.4 (–17– 5)	1 ± 1.7 (–8–3)	<0.01
Outliers > 5°	15 (55.6%)	1 (3.7%)	<0.01
Implanted in:			
Planned angle	0	13 (48.1%)	n.a.
Varus alignment	20 (74.1%); 8.8 ± 4.3°	4 (14.8%); 3.5 ± 3.3°	n.a.
Valgus alignment	7 (25.9%); 3.4 ± 1.3°	10 (37.0%); 1.4 ± 0.7°	n.a.
Deviation analyzing different bone morphologies:			
Physiological	7.0 ± 3.6°	1.4 ± 2.8°	<0.01
Osteoarthritis	8.1 ± 4.7°	0.6 ± 0.5°	<0.01
SCFE	7.0 ± 5.0°	1.1 ± 1.1°	<0.01
**Axial plane**			
Deviation (°)	5.1 ± 3.1 (–12–9)	3.9 ± 2.9 (–8–10)	0.13
Outliers > 5°	12 (44.4%)	8 (29.6%)	0.26
Implanted in:			
Planned angle	3 (11.1%)	1 (3.7%)	n.a
Anteversion	12 (44.4%); 6.0 ± 2.2°	20 (74.1%); 4.4 ± 2.8°	n.a.
Retroversion	12 (44.4%); 5.4 ± 3.2°	6 (22.2%); 2.8 ± 2.8°	n.a
Deviation analyzing different bone morphologies (°):			
Physiological	5.0 ± 3.7	2.5 ± 1.7	n.a.
Osteoarthritis	5.8 ± 1.9	6.3 ± 2.6	n.a.
SCFE	4.4 ± 3.6	2.8 ± 2.5	n.a.
**Notching**	8 (29.6%)	0	<0.01

Mean ± standard deviation (range). Deviation describes the difference between planned postoperative stem shaft angle.

For all three morphologic synthetic femur types, similar rates in deviations in the frontal plane were observed in the conventional (physiological 7.0 ± 3.6°, osteoarthritis 8.1 ± 4.7°, SCFE 7.0 ± 5.0°) and CAS (1.4 ± 2.8°, 0.6 ± 0.5°, 1.1 ± 1.1°) groups in favor of navigation.

### Notching and oversizing

**Figure 6:**
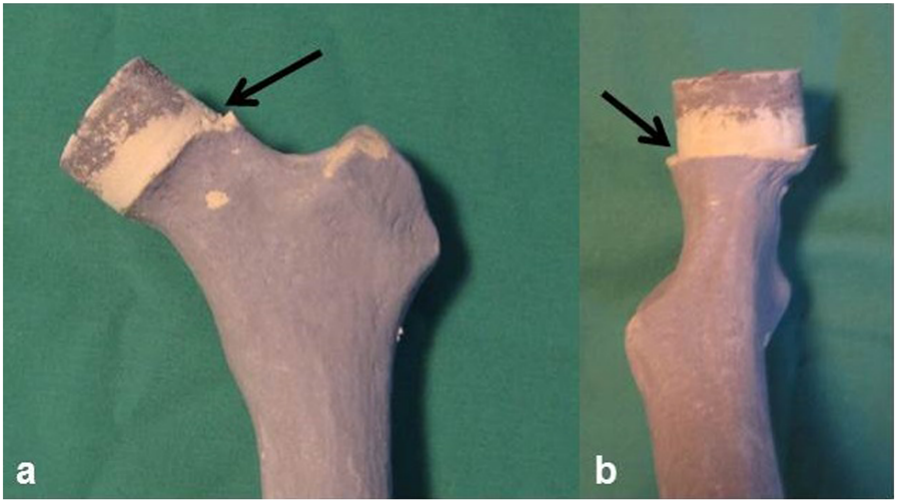
**Notching events in the superior (a) and posterior (b) quadrant (arrows).**

As presented in Table 3, notching was reduced by 100% in the CAS group as compared to the conventional group (n = 0, 0% vs. n = 8, 29.6%; *P* = 0.01). Superior (Figure 6a) notching occurred in 5 (18.5%), whereas posterior, inferior (Figure 6b), and anterior-superior notching was observed in 1 (3.7%) case each. Surgeon A generated most notching events (n = 5, 55.6%), followed by surgeon B (n = 2, 22.2%), and surgeon C (n = 1, 11.1%). A regression analysis demonstrated that only CAS (*P* <0.01) and the most experienced surgeon (*P* <0.01) reduced the rate of notching events. Notably, in all operations, the navigation software issued warnings for at least one quadrant when using the planned prosthesis size (Table 4). The software generally recommended a 7 ± 3.6 mm larger femoral component than was planned (Table 5). Surgeon A achieved the least average oversizing recommendation (4.22 ± 1.2 mm) followed by surgeon C (6.9 ± 2.3 mm) and surgeon B (10.0 ± 4.0 mm) as compared to the planned preoperative femoral component size. For the SCFE synthetic femur, the component size recommended by the software was 9.8 ± 4.2 mm larger as compared with the planned size. Similarly, for both other femur types (physiological 5.8 ± 2.3 mm and osteophytes 5.6 ± 2.4 mm) smaller prosthesis sizes were recommended by the CAS system.

**Table 4:** **True notching site when prepared with the conventional jig**

**Notching**	
**Location**	
Superior	5 (18.5%)
Inferior	1 (3.7%)
Posterior	1 (3.7%)
Superior-anterior	1 (3.7%)

**Table 5:** **Differences between planned femoral component size and component size recommended by the navigation software (mean ± SD, range)**

**Oversizing (mm)**	
Overall mean (range)	7.0 ± 3.6 (2–16)
**Surgeon**	
A	4.2 ± 1.2 (2–6)
B	10.0 ± 4.0 (4–16)
C	6.9 ± 2.3 (4–10)
**Bone morphology**	
Physiological	5.8 ± 2.3 (4–10)
Osteoarthritis	5.6 ± 2.4 (2–10)
SCFE	9.8 ± 4.2 (4–16)

### Procedure time and learning curve

The procedure time was increased in CAS group as compared to conventional group for each surgeon (overall mean ± SD, 18 ± 6 min vs. 14 ± 5 min; *P* = 0.028, Table 3) with the most inexperienced surgeon requiring the longest time for both CAS and the conventional procedure (Table 6).

**Table 6:** **Deviation between planed and postoperative angles as well as outliers exceeding 5° evaluating each surgeon, comparing computer-assisted surgery (CAS) vs. the conventional group (mean ± SD)**

	**Surgeon A**		**Surgeon B**		**Surgeon C**	
	**CAS**	**Conventional**	**CAS**	**Conventional**	**CAS**	**Conventional**
Time	24 ± 5 min	19 ± 6 min	15 ± 3 min	12 ± 3 min	14 ± 2 min	12 ± 3 min
**AP plane**						
Deviation (°)	1.0 ± 1.1	11.1 ± 4.4	1.6 ± 2.7	3.6 ± 1.5	0.6 ± 0.5	7.4 ± 3.1
Outliers	0	8 (88.9%)	1 (11.1%)	1 (11.1%)	0	6 (66.7%)
**Axial plane**						
Deviation (°)	5.2 ± 3.1	5.8 ± 3.9	3.2 ± 2.8	4.1 ± 2.9	3.1 ± 2.5	5.3 ± 2.6
Outliers	5 (55.6%)	4 (44.4%)	2 (22.2%)	4 (44.4%)	1 (11.1%)	4 (44.4%)
**Notching**	0	5 (55.6%)	0	2 (22.2%)	0	1 (11.1%)

SCFE, Slipped capital epiphysis.

Independent of the use of CAS, no learning curve was observed regarding precision, outlier rate, notching, and oversizing. However, a trend for a decrease in procedure time was observed for both surgeon A and B during the learning curve (Table 1).

## Discussion

HR is a demanding technique associated with specific complications caused by, e.g., imprecise positioning of the femoral or acetabular component [4,6,13-15]. Periprosthetic femoral neck fracture is the most common early complication occurring in up to 3% of the cases [3,16-18]. Shimmin et al. showed, in a series of 50 early neck fractures, that 72% of the femoral components were positioned in >5° varus alignment to the physiological CCD angle, that in 47% a superior notching was observed, and that in 36% both events occurred [18]. Evaluating the precision in the AP plane we observed a significant reduction in the overall deviation between postoperative and planned stem shaft angle using CAS (1 ± 1.7°) in comparison to the conventional procedure (7.4 ± 4.4°). Two preclinical studies using CAS without conventional control group showed deviations of 0.6° and 1° between the postoperative and the planned stem shaft angles [19,20]. In our study, precision was improved by CAS as compared to conventional technique (3.9 ± 2.9° vs. 5.1 ± 3.1°, *P* = 0.13) in the axial plane.

Using CAS dramatically reduced the overall number of outliers by 97% in the present study. Only one (4%) outlier occurred in the CAS group as opposed to 15 (56%) outliers in the control group. This finding is in line with other studies demonstrating the potential to reduce or even prevent positional outliers. For instance, in a retrospectively evaluated case series of 139 patients treated with HR using CAS (n = 51) or conventional method, Ganapathi et al. observed postoperative vs. planned SSA deviations exceeding 5° in 33 of 88 patients (38%) in the conventional group in contrast to no such case in the CAS group [12]. Similar findings were published by Resubal and Morgan: in their study, none vs. 31 of 131 (24%) outliers occurred in the CAS and conventional group, respectively [21]. A cadaver study performed by Davis et al. showed that surgeons tend to implant the femoral cap in varus orientation with a deviation up to 15° when using the manual jig, whereas this deviation was reduced to a maximum of 8° by the use of CAS [22,23]. Furthermore, we previously compared the same CAS system and conventional free-hand technique as evaluated in the present study in a prospectively randomized clinical trial and found that CAS significantly reduced the rate of outliers with five or more degrees of absolute deviation (4/37 vs. 12/38, respectively, Fisher’s exact *P* = 0.047) [23]. In addition, that study demonstrated a reduced incidence of varus outliers exceeding 5° by CAS (0/37 vs. 5/38 for CAS and control group, respectively, *P* = 0.054). In the current study, all Sawbone® samples were evaluated in a blinded manner by the three participating surgeons of differing levels of experience after intervention in respect to notching of the cortical head neck junction of the femur during intervention. CAS prevented notching in all cases, whereas 8 (29.6%) notching events were observed in the conventional group. Especially, the most inexperienced surgeon profited from CAS in terms of reduction in notching events. Ganapathi et al. [12] and Resubal and Morgan [21] showed similar findings, i.e., 4/88 (4.5%) and 3/131 (2.3%) notching events occurred in the conventional as opposed to none in the CAS group.

Contrary to the studies mentioned above, we performed a more precise examination of the femur. Postoperative evaluation of patients with X-ray has a potential risk of imprecision due to a more variable positioning of the patient during X-ray. Because of the X-ray racks for the Sawbones® we achieved projections exactly in the same pre- and postoperative position. Furthermore, both groups (conventional and CAS) are better comparable using the same Sawbone® morphologies as compared to high inter-individual variations *in vivo*.

With already minimal cortical bone damage being considered as notching and ambitiously having chosen a small sized femoral component in the present study, CAS showed a positive effect on both precision and reduction of outliers.

In addition to these encouraging confirmatory effects of CAS on femoral HR component orientation, the design of the present study allowed us to perform a comparative evaluation of the influence of CAS in terms of the surgeons’ skills. When analyzing the three participating surgeons individually, we observed that most notching events (56%) and outliers (89%) were generated by the surgeon who was at the beginning of his learning curve using the conventional technique. In contrast, that surgeon accomplished femoral head preparation without any outliers or notching events by use of CAS. This trend clearly demonstrates the benefit of CAS for unexperienced surgeons in the context of femoral HR component positioning. In a retrospective study of their first 550 cases of HR, Marker et al. reported periprosthetic fracture rates of 22% for the first 50 cases, of 2% for the following 50 cases, and of only 0.2% for the remaining 450 cases, thereby underlining the significance of a learning curve effect in HR [24]. Taking into account this suggested learning curve and the most common causes of periprosthetic femoral neck fracture, i.e., varus positioning increasing localized strain in the femoral neck on the one hand and superolateral notching reducing resistance to fracture on the other hand, an increased rate of femoral neck fractures is supposedly prevented for the most inexperienced surgeon in the clinical setting.

In the present study, the two more experienced surgeons B and C achieved a significant (1.6 ± 2.7° vs. 3.6 ± 1.5°; *P* <0.1, and 0.6 ± 0.5° vs. 7.4 ± 3.1°; *P* <0.01) increase in positional precision by the use of CAS, even though to a lesser extent than surgeon A (1.0 ± 1.1° vs. 11.1 ± 4.4°; *P* <0.01). A significant decrease of notching events was only observed when CAS was used or when surgeon C operated (*P* <0.01).

Only few studies have evaluated the influence of CAS on surgeons with different levels of experience. A Sawbones® study by Cobb et al. compared the deviations between planned and performed SSA during HR femoral component positioning by medical students [25]. In the conventional group, with either conventional or computed tomography planning, mean positional outliers of 23 ± 6° and 22 ± 7° occurred as contrasted by a significantly reduced mean deviation of 8 ± 2° in the CAS group (*P* <0.002). Seyler et al. reported on a retrospective study comparing cases of HR performed with CAS by two fellows, one with surgical experience of more than 250 conventional, free-hand HR and the other with more than 40 conventional HR and a group of senior residents at the beginning of their learning curve under close supervision by faculty [26]. Using CAS, both fellows and the group of residents produced comparable, low deviations between postoperative and planned SSA (0.9° ± 1.1; 1.5 ± 0.9; 1.2 ± 1.4). Interestingly, Olsen et al. [27], comparing the first 20 with the following 80 cases of their first 100 consecutive HR cases using navigation, found no significant learning curve effect with regard to femoral component placement, but a decrease in procedure time (26.8 min vs. 16.9 min, *P* = 0.002). In the current study, we did not observe any inter-surgeon difference in the positional deviation rates using CAS (Table 6). Contrary to the work by Cobb et al. [25], we used postdoc surgeons with different levels of experience more likely reflecting the clinical situation. The use of redundant bone morphologies in each group enabled us to make a more favorable inter-surgeon as well as inter-procedure comparison as compared with the clinical situation. Interestingly, we did not identify learning curves with regard to component placement precision or operative time.

There are still certain limitations to femoral component positioning with CAS. Although the scientific community is still uncertain on the exact position in the antetorsion of the femoral component, Pito et al. described larger axial vs. frontal deviations (mean ± SD, 3.4° ± 2.7–4.1 vs. 0.6° ± 0.4–0.7) between planned and postoperative femoral component orientation using CAS [18]. In line with these findings, we observed a mean axial deviation of 3.9° ± 2.9. The deviation seems to be less than in conventional placement, but is still not comparable to the precision in the AP projection.

At the same time, the navigation software investigated in the present study tended to over assess the femoral component size. This seemed to be dependent on the severity of deformation of the femoral head and neck as well as on surgeon-related variables, e.g., intraoperative virtual positioning of the HR component. Although the smallest component measured by hand was used, no notching events occurred in the CAS group. If the surgeons stringently followed the software using larger than possible femoral components, they would have run the risk of excessive acetabular bone reaming, which is contradictory to the bone-conserving philosophy of HR.

There are some relevant limitations of the study. One aspect is the fact that, due to logistic reasons, we only evaluated one particular CAS system as well as only one type of conventional jig. Future studies should address the issue of comparing different CAS-based and alternative conventional systems for HR femoral component placement. Furthermore, although greatest efforts were undertaken to mimic the exposure of the femoral bone from a posterolateral approach, the experimental setting still offered an easier spatial orientation in relation to the bony landmarks as compared to the intraoperative situs. Due to no muscle or tendon insertion in the sawbones, the orientation with the aiming device as well as the digitalization of the femoral head may have been easier.

## Conclusions

All surgeons achieved increased precision and less outliers when operating with CAS with the conventional technique. Notching was entirely prevented when using CAS. In particular, the most inexperienced surgeon profited by the use of CAS. We found no learning curve in respect of outliers and precision of component positioning. We observed excellent precision in implant positioning in the frontal plane for all bone morphologies using CAS. However, considering the axial plane we found a large variation in precision. In summary, imageless computer-assisted navigation has shown to be a valuable tool in our preclinical study to improve implant positioning in HR for surgeons at any stage of their learning curve. Therefore, further effects of CAS as a tool for implant positioning in HR in a clinical setup as well as the actual clinical relevance of improved precision need to be addressed in future studies.

## Abbreviations

3D: Three-dimensional
AP: Anterior-posterior
CAS: Computer assisted surgery
CCD: Centre collum diaphyseal
HR: Hip resurfacing
SCFE: Slipped capital epiphysis
SSA: Stem shaft angle
THR: Total hip replacement
